# PloverDB: a high-performance platform for serving biomedical knowledge graphs as standards-compliant web APIs

**DOI:** 10.1093/bioinformatics/btaf380

**Published:** 2025-06-28

**Authors:** Amy K Glen, Eric W Deutsch, Stephen A Ramsey

**Affiliations:** School of Electrical Engineering and Computer Science, Oregon State University, Corvallis, OR 97331, United States; Institute for Systems Biology, Seattle, WA 98109, United States; School of Electrical Engineering and Computer Science, Oregon State University, Corvallis, OR 97331, United States; Department of Biomedical Sciences, Oregon State University, Corvallis, OR 97331, United States

## Abstract

**Summary:**

Knowledge graphs are increasingly being used to integrate heterogeneous biomedical knowledge and data. General-purpose graph database management systems such as Neo4j are often used to host and search knowledge graphs, but such tools come with overhead and leave biomedical-specific standards compliance and reasoning to the user. Interoperability across biomedical knowledge bases and reasoning systems necessitates the use of standards such as those adopted by the Biomedical Data Translator consortium. We present PloverDB, a comprehensive software platform for hosting and efficiently serving biomedical knowledge graphs as standards-compliant web application programming interfaces. In addition to fundamental back-end knowledge reasoning tasks, PloverDB automatically handles entity resolution, exposure of standardized metadata and test data, and multiplexing of knowledge graphs, all in a single platform designed specifically for efficient query answering and ease of deployment. PloverDB increases data accessibility and utility by allowing data providers to quickly serve their biomedical knowledge graphs as standards-compliant web services.

**Availability and implementation:**

PloverDB’s source code and technical documentation are publicly available under an MIT License at github:RTXteam/PloverDB, archived on Zenodo at doi:10.5281/zenodo.15454600.

## 1 Introduction

Knowledge graphs (KGs) have emerged as useful abstractions for integrating heterogeneous biomedical data, making them convenient substrates for data mining and other computational reasoning techniques. The way in which such knowledge graphs are exposed for use varies; some graphs are only available for flat-file download, while others are exposed via a web application programming interface (API) or user interface, often backed by a general-purpose graph database management system such as Neo4j (github:neo4j/neo4j). Naturally, a knowledge graph’s ultimate utility is heavily influenced by the convenience, speed, and reliability of its mode of access.

The National Center for Advancing Translational Sciences (NCATS) Biomedical Data Translator (Fecho *et al.* 2022) (abbreviated as “Translator”) is a distributed knowledge graph-based computing system that integrates and reasons across disparate biomedical data to answer questions like *“What drugs might be repurposed to treat Fanconi anemia?”* The Translator Consortium has defined data standards for biomedical knowledge graphs, including (i) the *Biolink Model*, a standard schema and semantic layer for biomedical KGs (Unni *et al.* 2022), and (ii) *TRAPI*, a standard JavaScript Object Notation (JSON) web API format for biomedical KGs (github:NCATSTranslator/ReasonerAPI).

Within Translator, knowledge graphs are exposed via TRAPI-compliant web APIs that can answer single-edge or “one-hop” pattern-matching queries, such as *Acetaminophen—interacts_with*→*Protein?* (see Sec. Query structure), and in doing so, perform certain fundamental reasoning tasks like transitive chaining of concept subclass relationships (see Sec. Built-in reasoning). Such KG APIs can then be used by reasoning systems, such as Translator reasoning agents (e.g. Callaghan *et al.* 2023, Glen *et al.* 2023, and github:ranking-agent/aragorn) or large language model-based chain-of-thought reasoning systems, to answer larger queries.

As Translator’s requirements for interoperability and graph-based reasoning illustrate, transitioning from a flat-file version of a knowledge graph to a deployed, standards-compliant web API is not a trivial task. The KG owner must (i) canonicalize their graph (i.e. identify and unify semantically identical concept nodes); (ii) import it into some sort of database platform [such as Neo4j (Morton *et al.* 2019, Wood *et al.* 2022)]; (iii) convert incoming TRAPI queries into queries runnable on the back-end database system; (iv) encode various semantic and other fundamental reasoning tasks such as transitive chaining of subclass relationships; (v) ensure that answers are transformed back into TRAPI format; (vi) compute a meta knowledge graph and test triples; and (vii) deploy the entire service as a performant web API, even in the face of heavy concurrent load.

Previous work on web-accessible hosting frameworks for biomedical knowledge graphs includes Plater (github:TranslatorSRI/Plater), a tool for exposing a Neo4j-hosted Biolink-compliant KG as a TRAPI web API, which can be combined with Babel (github:TranslatorSRI/Babel) and ORION (github:RobokopU24/ORION, part of the ROBOKOP (Morton *et al.* 2019) software stack) to achieve entity resolution and KG formation/import, respectively. Another tool, BioThings Software Development Kit (Lelong *et al.* 2022), can dynamically create a Biolink-compliant API for a KG according to a custom data parser, which can then be exposed as a TRAPI API by BioThings Explorer (Callaghan *et al.* 2023).

Despite previous efforts, there is a need for a platform that allows data owners to easily deploy Biolink-compliant flat-file knowledge graphs as TRAPI web APIs that (i) is *comprehensive* in terms of back-end reasoning (e.g. entity resolution, concept subclass chaining) and provided artifacts/endpoints (e.g. meta KG, test triples), (ii) is *highly performant*, and (iii) can be *independently* deployed with *minimal effort* upon KG content or knowledge representation changes. To fill this gap, we created PloverDB, an all-in-one platform for efficiently hosting and serving Biolink-compliant KGs as TRAPI web APIs. We describe PloverDB’s implementation and usage in the following sections.

## 2 Implementation

PloverDB is a fully in-memory Python-based platform for hosting and serving Biolink-compliant knowledge graphs as TRAPI web APIs, designed specifically for improved query speed and ease of deployment. Its design prioritizes ease of use and minimal configuration, enabling rapid deployment of KGs with minimal overhead. PloverDB was initially created in 2021 to replace Neo4j as the back-end database platform for the RTX-KG2 knowledge graph (Wood *et al.* 2022), but was generalized in 2024 to (i) act as a standalone, fully TRAPI-compliant web service and (ii) be compatible with any Biolink-compliant knowledge graph. (Our primary motivation for moving on from Neo4j was performance, in particular query speed; while semantic reasoning can be encoded in Neo4j queries, it tended to cause a notable performance drop in our experience. Convenience of deployment was another factor).

**Figure 1.**
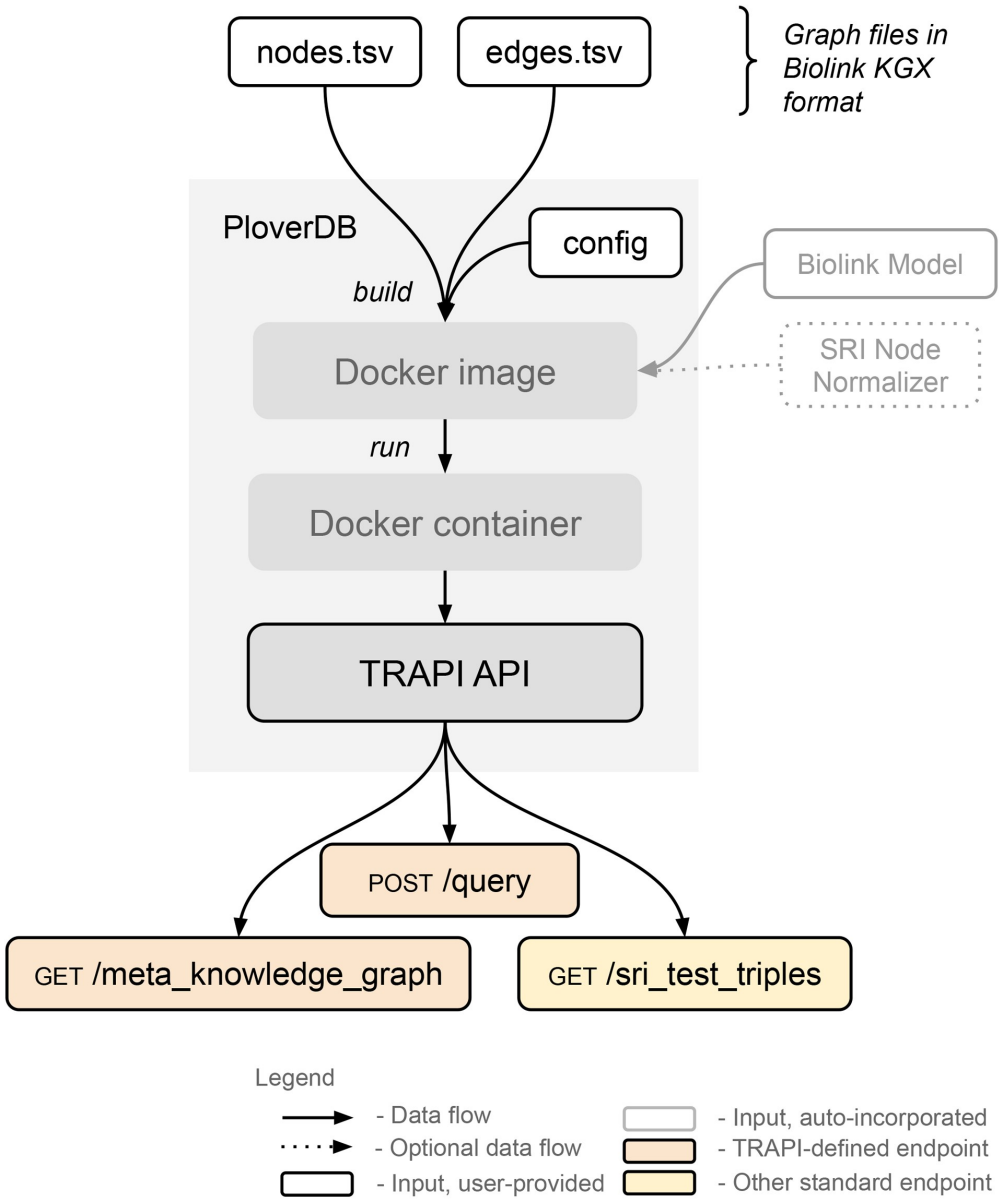
PloverDB’s high-level architecture, depicting how an input KGX-formatted knowledge graph is exposed as a TRAPI web API. Details are described in the main text.

PloverDB is Dockerized for convenient deployment, as depicted in Fig. 1.

During its Docker image build, PloverDB (i) downloads the Biolink-compliant KG nodes and edges files—which may be in either TSV or JSON Lines Knowledge Graph Exchange (KGX) format (github:biolink/kgx)—from user-specified public URLs provided in a JSON configuration file; (ii) loads the graph into memory; (iii) builds its core data structure and other indexes, utilizing the Biolink Model and Translator Node Normalizer (github:TranslatorSRI/NodeNormalization) service as applicable; (iv) optionally canonicalizes the graph; and (v) saves indexes to disk within the image. When a Docker container is then run from that image, PloverDB loads its core data structure and indexes into memory and starts a uWSGI/Flask server that exposes the graph via a TRAPI-compliant API, providing various endpoints (only standardized endpoints are depicted in Fig. 1).

PloverDB’s core data structure is essentially a nested adjacency dictionary in which nodes are mapped to their neighbors, organized by the categories of those neighbors and the types of edges that connect them to those neighbors. PloverDB does not use any intermediary database platforms such as Neo4j or SQLite, and instead stores the graph in Python data structures (dictionaries and sets). At runtime, multiple uWSGI workers (processes) share these read-only data structures to handle concurrent requests.

PloverDB is configured for a given knowledge graph via a single JSON config file, where users provide public URLs from which their KG files should be downloaded, can specify how node/edge properties should be represented in TRAPI attributes, and can control other options like graph canonicalization (see Sec. Usage).

### 2.1 Capabilities

PloverDB is designed to be a comprehensive platform for transforming a Biolink-compliant knowledge graph into a fully functioning TRAPI-compliant API. Its capabilities in terms of supported queries (Sec. Query structure), built-in reasoning (Sec. Built-in reasoning), ease of deployment (Sec. Simplified deployment), and performance (Sec. Performance) are detailed below.

#### 2.1.1 Query structure

PloverDB is designed to answer one-hop TRAPI queries, which are essentially graph-based pattern matching queries consisting of two nodes connected by a single edge (reasoning systems answer more complex queries by stitching together one-hop KG queries.). In such a “query graph,” nodes can be constrained by their concept (i.e. identifier) and semantic type (i.e. category) and edges can be constrained by their semantic type (i.e. predicate) and attributes (e.g. supporting publications, knowledge source, source type, etc.) Examples of such query graphs are provided in Section 1, available as supplementary data at *Bioinformatics* online.

#### 2.1.2 Built-in reasoning

Importantly, when PloverDB answers the queries defined in Section Query structure, it returns all knowledge subgraphs that fulfill the query graph not only *directly*, but also according to:

- the *hierarchies* of node and edge types according to Biolink,
- the *symmetry* of edge types according to Biolink,
- the *canonicity* of edge types according to Biolink,
- concept *equivalency*, and
- concept *subclass relationships* (via transitive chaining) present in the underlying graph or in a user-specified external source.

Of note, concept equivalency is determined either by (i) identifiers provided in an equivalent_identifiers (or similar) property on each node in the KG, or, in the absence of such a property, (ii) the Translator Node Normalizer service. PloverDB fulfills most of these reasoning tasks at build time and encodes them into its indexes for constant-time lookup at query time.

#### 2.1.3 Simplified deployment

PloverDB is designed to streamline deployment so that data owners can easily and independently update their service upon KG content/representation changes. Thus, it:

- is fully Dockerized and uses a standard Debian Linux-based base image from the public DockerHub registry (tiangolo/uwsgi-nginx-flask:python3.11),
- automatically constructs and exposes a TRAPI meta knowledge graph and corresponding test triples,
- allows multiple KGs to be served from the same PloverDB application, accessible at separate sub-endpoints (i.e. “multiplexing” of KGs),
- provides a built-in (authenticated) remote deployment mechanism, enabling data owners to remotely redeploy their service with minimal downtime,
- provides endpoints to aid remote debugging, that expose log and KG/code version information, and
- can (optionally) be registered in SmartAPI (Zaveri *et al.* 2017) using the TRAPI registration template (github:NCATSTranslator/ReasonerAPI/TranslatorReasonerAPI.yaml).

#### 2.1.4 Performance

PloverDB uses a fully in-memory architecture for efficient query answering. To quantify its performance, we conducted a controlled study comparing PloverDB to Plater, a previously mentioned functionally similar platform that uses Neo4j for its back-end, when hosting the RTX-KG2 knowledge graph. The study included 82 queries for which the platforms returned successful responses: 79 real-world, randomly selected queries and three hand-crafted queries, designed to address gaps in terms of answer size. We present highlights from this study below; full methodology details, results, and discussion for this study are provided in Section 2, available as supplementary data at *Bioinformatics* online.

**Figure 2.**
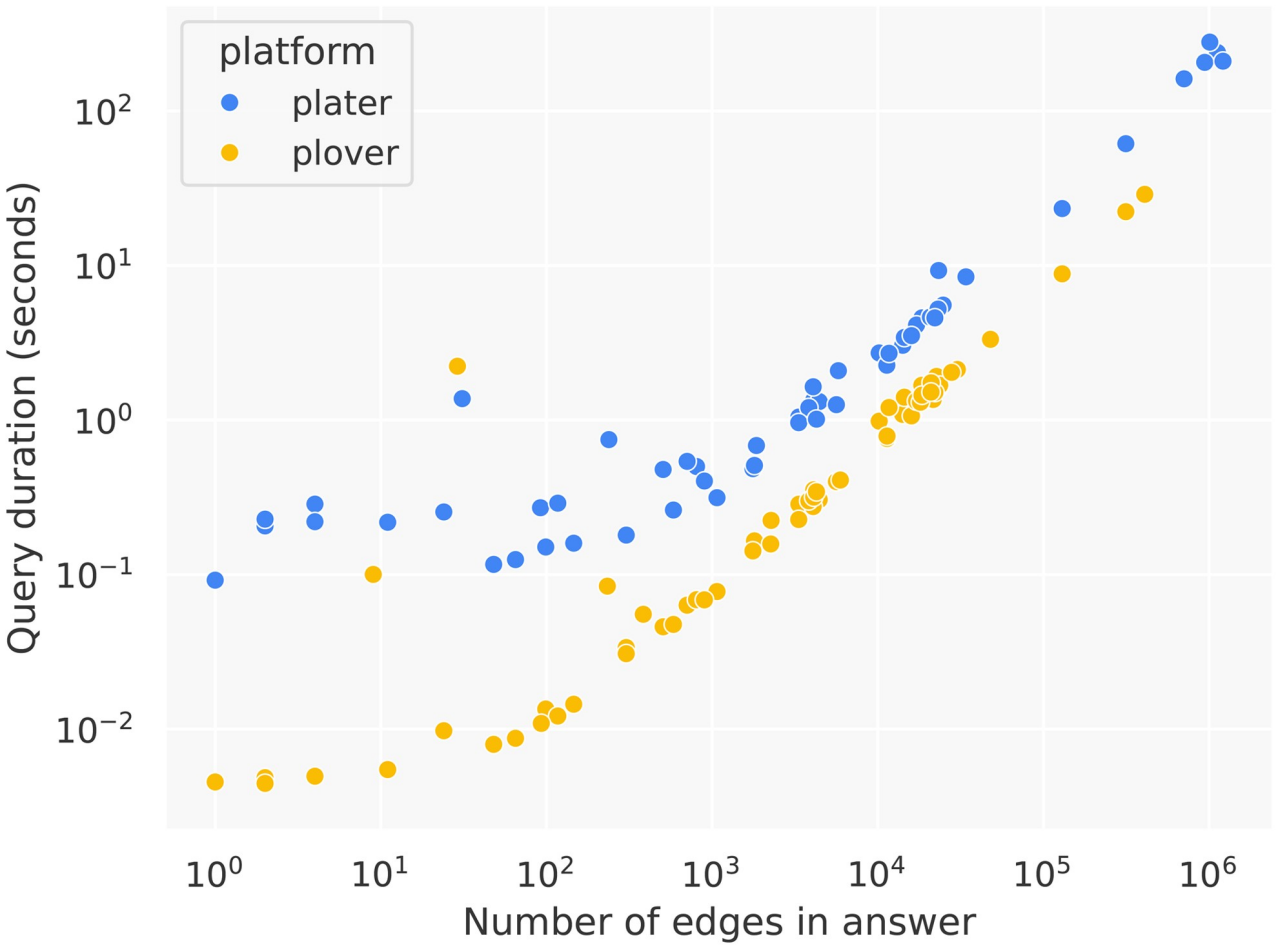
Query response time as a function of answer size for PloverDB versus Plater when hosting the RTX-KG2 knowledge graph, based on a set of 82 largely randomly selected real-world queries. Results are displayed on a log-log scale for better visibility; on a linear scale, both platforms form straight lines, but Plater has a three-times steeper slope than PloverDB (see Fig. 2, available as supplementary data at *Bioinformatics* online). Details on the included queries are provided in Sections 2.1.3 and 2.2.1, available as supplementary data at *Bioinformatics* online.

In this controlled performance study, both platforms’ response times appeared asymptotically linear in terms of the number of edges in the query answer, but Plater had a three-times steeper slope than PloverDB, at 0.022 versus 0.007 s per 100 answer edges. Figure 2 visualizes these results on a log-log scale; the magnitude of difference in query speed between the two platforms was greater for smaller queries, suggesting that Plater has higher overhead. The proportion of time Plater spent on Neo4j (as opposed to “wrapper” code around database calls) ranged from 80% to 99%, and showed a negative relationship with query answer size (Fig. 7, available as supplementary data at *Bioinformatics* online), suggesting Plater’s overhead arose from Neo4j.

PloverDB was 14-times faster than Plater on average across the 51 randomly selected queries for which the two platforms returned identical answer sets (Table 1, available as supplementary data at *Bioinformatics* online). In a ramped load test to extreme conditions, PloverDB averaged 4.5-times higher throughput and 9-times faster response time than Plater (Section 2.2.4, available as supplementary data at *Bioinformatics* online).

PloverDB’s speed and low overhead observed in this study come with a trade-off; its in-memory design means that it is relatively memory-hungry, using 79 GiB of system memory at rest when hosting RTX-KG2.8.4c (7 million nodes and 27 million edges) versus only 5 GiB for Plater. While PloverDB’s memory consumption depends heavily on the hosted graph’s content (e.g. the size and number of node/edge attributes) and configuration choices, our observations from this study and real-world deployments suggest that PloverDB tends to use roughly 3–5 GiB of memory per million edges.

## 3 Usage

Full technical details on how to use and deploy PloverDB are provided in GitHub at github:RTXteam/PloverDB. Assuming that Docker has been installed on the host computer and the PloverDB code repository has been cloned into the local file system, deploying PloverDB is simply a matter of:

- editing PloverDB/app/config.json for the user’s KG, and
- running bash PloverDB/run.sh, which builds and starts the Dockerized PloverDB service.

The PloverDB repository includes a template for the config.json file. Most significantly, users must update the nodes_file and edges_file slots with URLs from which PloverDB can download the graph’s nodes and edges files at image build time. These files must adhere to Biolink KGX (github:biolink/kgx) TSV or JSON Lines format. Users may also specify how node/edge properties should be represented as TRAPI Attributes using the trapi_attribute_map slot. Explanations for all config.json properties are available in the PloverDB GitHub repository, as well as steps to deploy PloverDB in a production setting, including TLS certificate installation.

The fact that the config.json file is tracked in the PloverDB software repository makes configuring continuous deployment straightforward; users can set up continuous deployment on a fork of the repository using a third-party tool, or they can use PloverDB’s built-in remote deployment mechanism, which allows users to deploy changes by submitting an authenticated /rebuild request to the PloverDB web service. Multiplexing KGs is a matter of adding an additional JSON config file for each KG to be served. PloverDB will automatically build and expose a TRAPI endpoint for each such KG, accessible at the endpoint_name specified in its configuration file.

At the time of writing, PloverDB is used to host and serve five different knowledge graphs for the Translator system: RTX-KG2 (Wood *et al.* 2022) (kg2cploverdb.transltr.io/kg2c), Microbiome KG (Goetz *et al.* 2024) (multiomics.transltr.io/mbkp), Multiomics KG (Qin *et al.* 2024) (multiomics.transltr.io/mokp), Clinical Trials KG (Qin *et al.* 2024) (multiomics.transltr.io/ctkp), and Drug Approvals KG (Qin *et al.* 2024) (multiomics.transltr.io/dakp), ranging in size from 5000 nodes and 45 000 edges to 7 million nodes and 27 million edges.

## 4 Conclusion

PloverDB is a software platform for efficiently hosting and serving Biolink-compliant knowledge graphs as standardized TRAPI-compliant web APIs. PloverDB emphasizes simplicity and automation, allowing knowledge graph owners to focus on their data rather than the technicalities of exposing it. Its comprehensiveness in terms of built-in reasoning and ease of deployment significantly reduce the barrier of entry for exposing knowledge graphs for use by others, thereby enhancing their utility for advancing biomedicine.

## Supplementary Material

btaf380_Supplementary_Data
